# Blocking P2X purinoceptor 4 signalling alleviates cigarette smoke induced pulmonary inflammation

**DOI:** 10.1186/s12931-022-02072-z

**Published:** 2022-06-08

**Authors:** Sven Schneider, Irmgard Merfort, Marco Idzko, Andreas Zech

**Affiliations:** 1 Department of Pneumology, University Medical Centre Freiburg, Freiburg, Germany; 2 Department of Pulmonology, Internal Medicine II, Medical University of Vienna, Vienna, Austria; 3 Institute of Pharmaceutical Sciences, Albert-Ludwigs-University Freiburg, Freiburg, Germany

## Abstract

**Background:**

Chronic obstructive pulmonary disease (COPD) is associated with elevated ATP levels in the extracellular space. Once released, ATP serves as danger signal modulating immune responses by activating purinergic receptors. Accordingly, purinergic signalling has been implicated in respiratory inflammation associated with cigarette smoke exposure. However, the role of P2X4-signalling has not been fully elucidated yet.

**Methods:**

Here, we analysed the P2X4 mRNA expression in COPD patients as well as cigarette smoke-exposed mice. Furthermore, P2X4-signalling was blocked by either using a specific antagonist or genetic depletion of P2rx4 in mice applied to an acute and prolonged model of cigarette smoke exposure. Finally, we inhibited P2X4-signalling in macrophages derived from THP-1 before stimulation with cigarette smoke extract.

**Results:**

COPD patients exhibited an increased P2X4 mRNA expression in cells isolated from the bronchoalveolar lavage fluid and peripheral mononuclear cells. Similarly, P2rx4 expression was elevated in lung tissue of mice exposed to cigarette smoke. Blocking P2X4-signalling in mice alleviated cigarette smoke induced airway inflammation as well as lung parenchyma destruction. Additionally, human macrophages derived from THP-1 cells released reduced concentrations of proinflammatory cytokines in response to cigarette smoke extract stimulation when P2X4 was inhibited.

**Conclusion:**

Taken together, we provide evidence that P2X4-signalling promotes innate immunity in the immunopathologic responses induced by cigarette smoke exposure.

**Supplementary Information:**

The online version contains supplementary material available at 10.1186/s12931-022-02072-z.

## Background

About two decades ago, adenosine 5′-triphosphate (ATP) has been indicated to play a role in chronic obstructive airway diseases for the first time [1]. Released into extracellular space ATP serves as damage-associated molecular pattern (DAMP), which drives inflammation by facilitating recruitment as well as activation of various immune cell types via binding to purinergic receptors [2, 3]. Consequently, elevated levels of extracellular ATP in the bronchoalveolar lavage fluid (BALF) have been reported in chronic inflammatory diseases of the airways including asthma and chronic obstructive pulmonary disease (COPD) [4, 5]. Accordingly, mice exposed to cigarette smoke exhibited increased ATP concentrations in the BALF compared to air controls [6]. The increased ATP release upon inflammatory conditions in the airways has been attributed to epithelial cells and activated immune cells such as macrophages and neutrophils [2, 7].

According to their functional properties, the P2 receptors are classified into the ionotropic P2X and the metabotropic P2Y receptors [8]. Members of both subclasses have been implicated in COPD pathogenesis. For instance, P2X7 activation has been shown to promote pulmonary inflammation via IL-1ß mediated release of proinflammatory cytokines, chemokines and proteases [9, 10]. Accordingly, genetic ablation of *P2rx7* resulted in reduced IL-1ß production and alleviated cigarette smoke (CS)-induced airway inflammation in mice [11]. P2X7 shares high similarities in terms of sequences, expression patterns and physiological functions with P2X4 [12]. Interestingly, several studies suggest an interaction between these two P2X receptors [13–16]. However, although P2X7 has been implicated in CS-induced airway inflammation a potential role of P2X4 has not been elucidated yet.

During the last two decades much progress has been made in understanding the complex interplay of extracellular ATP and the pathophysiological processes in pulmonary inflammation [17]. However, many aspects about the role of purinergic signalling in chronic lung disorders like COPD remain elusive. Therefore, the aim of this study was to investigate the potential role of P2X4 in CS-induced airway inflammation as well as airway remodelling. Here we demonstrate that P2X4-signalling contributes to CS-induced airway inflammation by promoting innate immunity.

## Methods

### Human subjects

Individuals with COPD and healthy never-smokers (Additional file 1: Tables S1 and S2) were recruited at University Hospital Freiburg, Germany. Patients and healthy volunteers gave their written informed consent to use the obtained biologic samples for research. The analysis of the samples was approved by the local ethics committee (EC Nr. 06.2006 & 10.2006).

### Mice

C57BL/6N (6–8 weeks old) mice were purchased from Charles River (Sulzfeld, Germany). The *P2rx4*-deficient (MGI: 3665297) mouse was originally designed in Francois Rassendren’s lab at the Centre National de la Recherche Scientifiqu in the Department of Molecular Pharmacology as previously described [18]. *P2rx4*-deficient animals were crossed back in C57BL/6 N background and bred under specific pathogen-free (SPF) conditions in the animal facility of Freiburg University. Male, 8–10 weeks old mice were used for all the experiments. For experiments including solely wild type mice, the treated animals and the corresponding controls were littermates. Experiments on *P2rx4*-deficiency included *P2rx4*-deficient mice and wild type controls from separate nests. All mice experiments were approved by the local ethics committee [G-12/106, G-14/108] and executed according to national law.

### Murine models of cigarette smoke-induced airway inflammation and P2X4 inhibition

A whole-body exposure chamber containing the mice was mechanically ventilated (7025 rodent ventilators, Ugo Basile, Biological Research Instruments, Comerio, Italy) with either the smoke from five commercial Marlboro Red cigarettes (10 mg of tar, 10 mg of carbon monoxide and 0.9 mg nicotine) diluted 1:10 with air or air alone for 20 min. Mice were exposed to CS of five subsequent lit cigarettes for 20 min daily on three consecutive days in an acute or on each of five consecutive days per week for four months in a chronic approach.

Intratracheal (i.t.) administration of 80 µl PBS containing 10 µM 5-(3-Bromophenyl)-1,3-dihydro-2H-benzofuro-[3,2-e]-1,4-diazepin-2-one dissolved in DMSO (Tocris, Bristol, UK) or 80 µl PBS with 0.05% DMSO as vehicle control was performed 30 min prior to every smoke exposure. The amount of 10 µM chosen was based on preliminary dosing experiments (data not shown) according to results from an ATP-induced current inhibition assay applied with transfected HEK293 cells [19]. To minimize cage effects mice within the same cage were randomly allocated to the different treatment groups of the corresponding experiment.

### Bronchoalveolar lavage fluid (BALF)

First, the trachea of sacrificed mice was penetrated with a cannula. Subsequently, the lung was flushed with 0.75 ml PBS (Gibco, Thermo Fisher Scientific, Germany) supplemented with 0.5 mM EDTA (Sigma-Aldrich, Taufkirchen, Germany) four times. Every first flush was collected in a separate tube and the corresponding supernatant was later used for cytokine measurements via enzyme-linked immunosorbent assay (ELISA). BAL cells from individual mouse were pooled and used for differential cell counting via flow cytometry.

### Histology

After fixing the lungs with 10% formalin (Merck, Darmstadt, Germany) for 48 h, they were embedded in paraffin. The prior cut 7 µm lung sections were dewaxed and rehydrated using xylene and a series of decreasing alcohol concentrations. Finally, hematoxylin and eosin were applied to stain lung sections (Carl Roth, Karlsruhe, Germany).

Forty histological fields were evaluated both vertically and horizontally to determine the average interalveolar distance (mean linear intercept: Lm). Counting was performed independently by two members of the lab. The mean Lm and postfixation lung volume were used to calculate the internal surface area of the lungs (ISA) [20].

### Flow cytometry

First, BALF cells were washed in PBS containing 0.5% BSA and 0.01% NaN_3_ and counted. To avoid nonspecific binding cells were incubated with an unlabeled anti-CD16/32 antibody (AB_467133) before adding a mix containing anti-Gr-1 FITC − (AB_465314), anti-F4/80 PE − (AB_465923), anti-Cd11c APC − (AB_469346), anti-CD3e PE-Cy7 (AB_469572) -labeled antibodies (eBioscience, San Diego, CA). Flow cytometry was performed using a FACSCalibur flow cytometer (BD Biosciences, San Jose, CA). Data were analyzed using FlowJo v10 (TreeStar, Ashland, OR) software. Different cell types were identified with the aid of differential marker expression (CD3/B220 + CD11c − Lymphocytes, F4/80 + CD11c + macrophages and Gr-1 + CD3/B220 − CD11c − neutrophils) and via forward and side scatter positioning.

### Quantitative real-time polymerase chain reaction (qPCR)

Murine lungs were incubated in RNAlater Stabilization Reagent (QIAGEN GmbH, Hilden, Germany) at 2–8° C overnight and frozen for long-term storage (− 20 °C). Total RNA from human cells or murine tissue was isolated using QIAzol lysis reagent (QIAGEN GmbH, Hilden, Germany). Chloroform was added to the lysates for separation. The aqueous phase containing the RNA was transferred before isopropanol was added for precipitation. The RNA pellet was washed two times with 70% Ethanol for the subsequent two washing steps, and eventually dissolved in nuclease-free water. 28S and 18S rRNA bands were evaluated after gel electrophoresis to confirm RNA integrity. After eliminating remaining genomic DNA via gDNA eliminator column (QIAGEN GmbH, Hilden, Germany), first strand cDNA synthesis kit (Thermo Fisher Scientific GmbH, Germany) was used to synthesize cDNA. The qPCR mixes containing Takyon mastermix (Eurogentec, Köln, Germany) were applied to a LightCycler 480 (Roche, Mannheim, Germany). Primer sequences are available in the Additional file 1. Gene expression was calculated using the following formula:

$${\text{expression}}\,{\text{relative}}\,{\text{to }}\beta 2{\text{m}} = 100 \times 2^{{ - \Delta {\text{Ct}}}}$$

### Cigarette smoke extract stimulation of macrophages derived from monocyte-like THP-1 cells

THP-1 cells were obtained from German Collection of Microorganisms and Cell Cultures GmbH (Braunschweig, Germany). Undifferentiated THP-1 cells were cultivated in RPMI-1640 medium (Gibco, Thermo Fisher Scientific, Germany) supplemented with 1% penicillin/streptomycin and 10% heat-inactivated fetal calf serum (FCS, Biocell Laboratories, Rancho Dominguez, CA) in 150 cm [2] culture flask (Corning Life Sciences, NY, USA) till a density of 3 × 10^7^ was reached. After adjusting to 1.5 × 10^6^ cells/well in a 6-well plate (Greiner Bio-One, Frickenhausen, Germany), cells were incubated with 100 nM phorbol 12-myristate 13-acetate (PMA) (Sigma-Aldrich, Taufkirchen, Germany) for three days to induce macrophage-like differentiation. Twenty-four hours before stimulation, the cells were detached by using 5 ml accutase (Sigma-Aldrich, Taufkirchen, Germany) for 45 min at 37 °C and adjusted to 2.5 × 10^5^ cells/well in a 24-well-plate (Greiner Bio-One, Frickenhausen, Germany). After letting the cells adhere for 2 h, the medium was removed and replaced with FCS-free medium for conditioning.

The 100% cigarette smoke extract (CSE) was prepared by infusing CS of one cigarette (Marlboro Red, 10 mg of tar, 10 mg of carbon monoxide and 0,9 mg nicotine) into 10 ml pre-warmed 37 °C RPMI medium using a 50 ml syringe, followed by sterile filtration through a 0.22 μm cellulose acetate sterilizing system (Corning Life Sciences, NY, USA). The CSE was used immediately after preparation. A 5% CSE stimulation is associated with smoking slightly less than one pack of cigarettes per day. To block P2X4-signalling, 10 µM 5-(3-Bromophenyl)-1,3-dihydro-2H-benzofuro-[3,2-e]-1,4-diazepin-2-one (5-BDBD) or vehicle (PBS with 0.05% DMSO) were added to the cell culture 30 min prior to CSE stimulation.

### Measurement of mediator concentrations

To determine the concentrations of inflammatory mediators including CXCL1/KC, MCP-1, IL-1ß, IL-6, neutrophil elastase, MMP-9 and TNFa, sandwich ELISAs was applied. Two replicates for standard as well as supernatant samples were measured and supernatant samples were diluted in the solvent of the standard protein if necessary. All ELISA kits were purchased from R&D Systems (Wiesbaden-Nordenstadt, Germany) and the procedure was performed according to the corresponding manufacturer’s kit manuals.

### Statistical analysis

Statistical tests were performed using Graphpad Prism v9 (GraphPad Software, San Diego, CA) and Microsoft Excel. All data was analysed for homoscedasticity using Levene’s and Brown-Forsythe test. The *P2RX4* expression in human cells and *P2rx4* expression in murine lung tissue were analysed using the unpaired *t*-test with Welch’s correction to adjust for unequal variance. The data concerning antagonist treatment were analysed Welch’s analysis of variance (ANOVA) with Dunnett-T3 post hoc test to correct for multiple comparisons. Data concerning *P2rx4*-deficincy were analysed using two-way ANOVA with Tukey’s post hoc test to correct for multiple comparisons. P ≤ 0.05 was considered statistically significant.

## Results

### COPD and cigarette smoke exposure are associated with an enhanced *P2RX4* expression

**Fig. 1:**
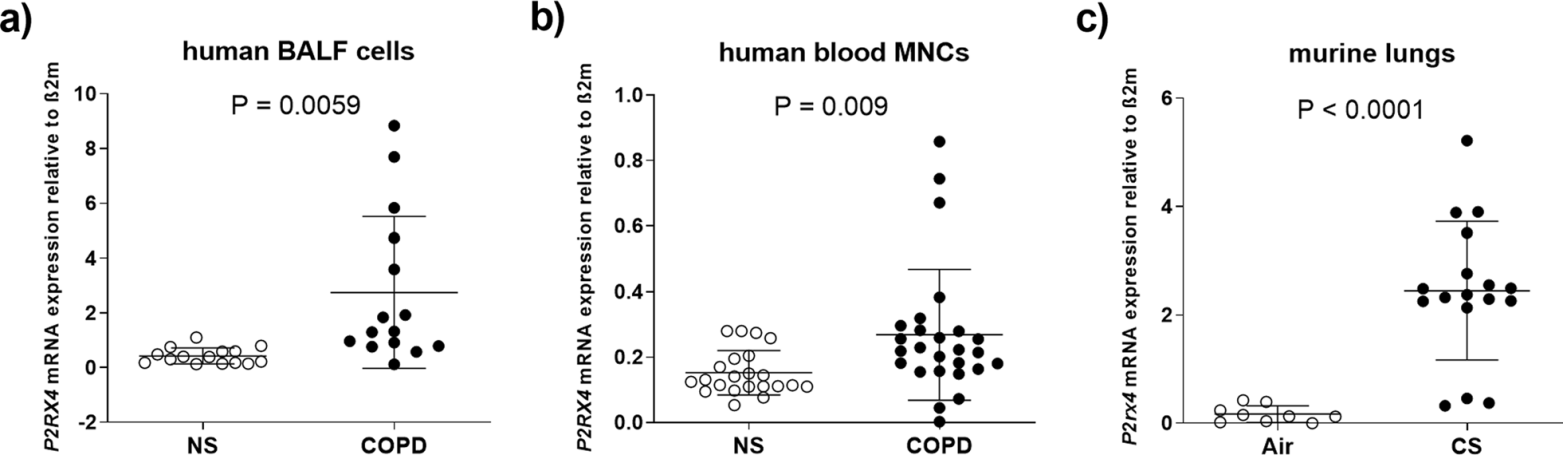
P2RX4 expression in murine lung tissue, human BALF and peripheral blood mononuclear cells. Relative *P2rx4* (murine)/*P2RX4* (human) expression determined via quantitative PCR in a) BALF cells and b) peripheral blood mononuclear cells obtained from COPD patients as well as in c) murine lung tissue isolated after cigarette smoke (CS) exposure for three consecutive days compared to non-smoking (Air/NS) individuals. P2X4 mRNA expression was quantified relative to ß2 microglobulin (ß2m) levels. mice: Air n = 9, CS n = 17; human BALF: n = 15 in both groups; human blood mononuclear cells (MNCs): never smokers n = 22, COPD n = 26. Each symbol represents individual subject. Graphs include mean with standard deviation unpaired *t*-Test with Welch’s correction was applied for group comparison. P is depicted in the corresponding graph and was considered statistical significant when ≤ 0.5

To investigate whether obstructive pulmonary disease (COPD) is associated with an altered *P2RX4* expression, the *P2RX4* expression in BALF cells as well as blood mononuclear cells (MNCs) of COPD patients was compared to never-smoking healthy individuals. Interestingly, COPD-patients showed an elevated *P2RX4* in both, BALF cells and MNCs (Fig. 1a, b). Similarly, lung tissue (Fig. 1c) as well as BALF cells (Additional file 1: Fig. S1), isolated from mice exposed to CS for three consecutive days, exhibited an increased *P2rx4* expression compared to the air-control animals.

In case of stratification by the Global Initiative for Chronic Obstructive Lung Disease (GOLD) classification into “GOLD III and IV” and “GOLD I and II” COPD patients, *P2RX4* expression was increased in BALF cells and blood MNCs of GOLD III and IV COPD patients while COPD patients classified as GOLD I and II showed no significant difference compared to never-smokers (Additional file 1: Fig. S2). Of note, the active smokers among the GOLD I and GOLD II COPD patients tended to exhibit a higher *P2RX4* expression compared to GOLD I and GOLD II ex-smokers. However, due to the exploratory character of this human study there are differences in known confounding factors including age and gender ratio between the COPD patients and the never-smoking healthy subjects (Additional file 1: Tables S1, S2).

### Blocking P2X4-signalling alleviates cigarette smoke-induced airway inflammation

**Fig. 2:**
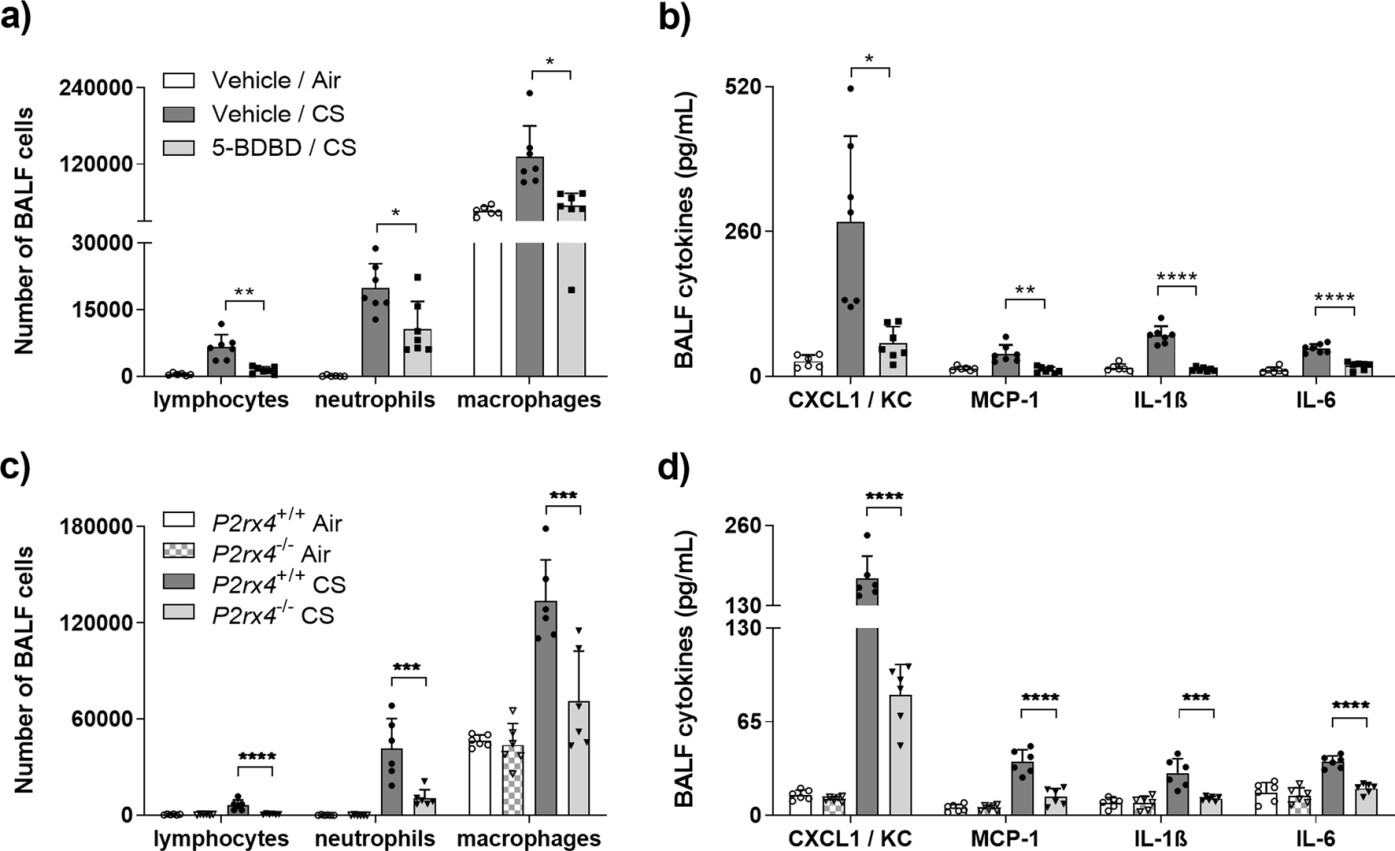
Inhibition of P2RX4-mediated signalling alleviates CS-induced airway inflammation in mice. Differential cell counts as well as concentrations of proinflammatory cytokines (CXCL1/KC, MCP-1, IL-1ß, IL-6) in the BALF of mice **a**, **b** treated with 10 µM 5-BDBD 30 min prior each daily smoke exposure, and **c**, **d** deficient for *P2rx4* (*P2rx4*^−/−^) after cigarette smoke (CS) exposure for three consecutive days compared to untreated (Vehicle/CS) or wild type (*P2rx4*^+/+^) animals, respectively. Data is presented as individual symbols (n = 6–7) and mean + SD. Treatment data was analysed applying Welch’s ANOVA corrected for multiple comparison using Dunett’s T3 test. Deficiency data was analysed applying two-way ANOVA corrected for multiple comparison using Tukey test. *P < 0.05, **P < 0.01, ***P < 0.001, ****P < 0.0001

To evaluate the role of P2X4 in CS-induced airway inflammation mice were treated with the selective P2X4 antagonist 5-BDBD i.t. prior daily CS exposure. Consequently, 5-BDBD administration caused an alleviated CS-induced acute airway inflammation compared to vehicle instillation in C57BL/6 N mice. This was evidenced by the reduction in numbers of BALF neutrophils, macrophages and lymphocytes (Fig. 2a) as well as concentrations of the proinflammatory cytokines CXCL1/KC, MCP-1, IL-1ß and IL-6 in the BALF supernatant (Fig. 2b).

For further confirmation, *P2rx4*-deficient mice were subjected to CS exposure on three consecutive days. Similar to 5-BDBD treatment, *P2rx4*-deficiency was associated with decreased differential cell numbers in the BALF compared to wild type control animals (Fig. 2c). Likewise, *P2rx4-*deficient mice exhibited reduced BALF concentrations of CXCL1/KC, MCP-1, IL-1ß and IL-6 (Fig. 2d). Interestingly, *P2rx4* deficiency was also associated with diminished IL-17 concentrations (Additional file 1: Fig. S3A). Altogether, the results suggested that P2X4-signalling promotes acute CS-induced airway inflammation.

### *P2rx4*-deficiency dampens cigarette smoke-driven emphysema development

Besides proinflammatory cytokines, *P2rx4*-deficiency was associated with a diminished release neutrophil elastase and MMP-9 (Additional file 1: Fig. S3B) after acute smoke exposure. Among other tissue-degrading proteases, MMP-9 and neutrophil elastase have been implicated in airway remodelling and emphysema development during COPD [21]. The reduction of these mediators in the BALF of *P2rx4*-deficient mice suggested that P2X4-signalling drives airway remodelling by facilitating protease release.

**Fig. 3:**
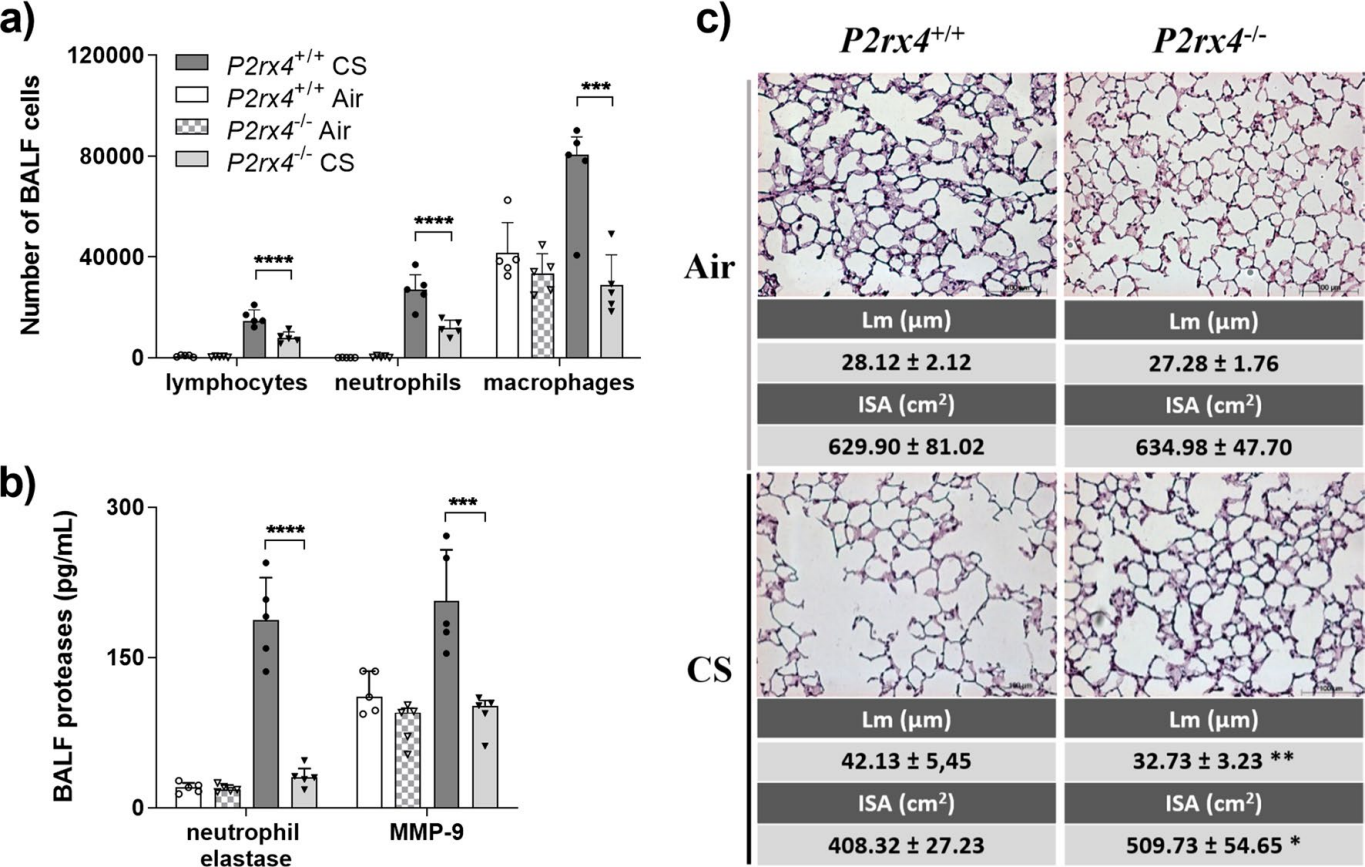
*P2rx4*-deficiency is associated with reduced airway inflammation and remodelling in response to regular cigarette smoke exposure for 4 months. **a** Differential cell count, and **b** concentrations of proteases in the BALF (ELA2, MMP-9) of *P2rx4*-deficient mice (*P2rx4*^−/−^) after exposure with the smoke of 5 cigarettes per day, five days a week for four months compared to wild type animals (*P2rx4*^+/+^). **c** Exemplary hematoxylin & eosin stained paraffin lung section (40 × magnification, 100 µm scale bar) used to quantify lung parenchyma destruction via calculating the linear mean intercept (Lm) and mean internal surface area (ISA). Mean Lm and ISA ± SDs are depicted under the corresponding histology pictures. BALF data is presented as individual symbols (n = 5) and mean + SD. Two-way ANOVA corrected for multiple comparison using Tukey test was applied for group comparison. *P < 0.05, **P < 0.01, ***P < 0.001, ****P < 0.0001

To evaluate the role of P2X4 in airway remodelling and emphysema development *P2rx4*-deficient mice were exposed to CS five days a week for four months. Analogous to the acute approach, *P2rx4*-deficiency was associated with diminished neutrophil, macrophage, and lymphocyte numbers (Fig. 3a) as well as concentrations proinflammatory cytokines (Additional file 1: Fig. S4), neutrophil elastase, and MMP9 (Fig. 3b) in the BALF compared to wild type controls. Furthermore, *P2rx4*-deficient mice were partially protected from lung parenchyma destruction evidenced by a reduced mean linear intercept (Lm) and increased mean internal surface area (ISA) (Fig. 3c).

### Inhibiting P2X4-signalling alleviates the release of proinflammatory cytokines by monocyte-derived macrophages in response to cigarette smoke extract stimulation

Airway inflammation as well as remodelling is driven by chronic pulmonary inflammation perpetuated by immune cells infiltrating the lung. Myeloid cells and lymphocytes have been shown to play a crucial role in COPD pathogenesis and both cell types, also express high *P2RX4* levels [22–24]. Since *P2RX4* is expressed in alveolar macrophages and shows an increased expression in MNCs isolated from COPD patients (Fig. 1b), we suggested monocyte-derived cells to play a pivotal role in P2X4-mediated effects in acute cigarette smoke-induced airway inflammation.

**Fig. 4:**
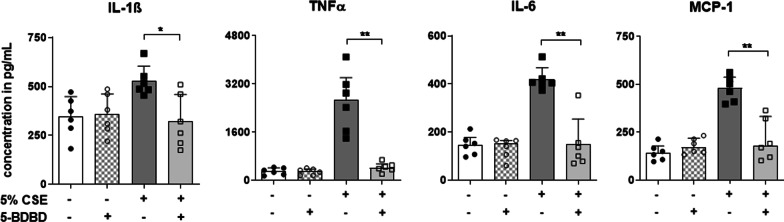
Blocking P2X4-signaling in THP-1 derived macrophages alleviates pro-inflammatory cytokine release in response to cigarette smoke extract stimulation. Concentrations of IL-1ß, TNFα, IL-6 and MCP-1 in the supernatant of THP-1 derived non-polarized macrophages treated with 10 µM of the specific P2X4-antagonist 5-BDBD 30 min prior to stimulation with 5% cigarette smoke extract compared to non-treated cells. Data is presented as individual symbols for 6 wells from three different experiments and mean + SD. Welch’s ANOVA corrected for multiple comparison using Dunett’s T3 was used for group comparison. *P < 0.05, **P < 0.01

To investigate the role of P2X4-signalling in myeloid cells, we assessed the secretion of proinflammatory cytokines by 5-BDBD treated human non-polarized (M0) macrophages derived from P2X4-expressing THP-1 cells [25, 26] in response to CSE stimulation. Indeed, 5-BDBD treated THP-1 derived macrophages secreted less TNFα, IL-1ß, MCP-1 and IL-6 compared to cells not pre-incubated with 5-BDBD (Fig. 4).

## Discussion

It has been clearly shown that purinergic signalling plays a role in airway inflammation in mice and in humans [2]. However, the particular contribution of the different receptor subtypes to the pathological mechanisms related to the inflammatory response have not been fully elucidated, yet. A previous study has implicated that P2X7 drives CS-induced airway inflammation by modulating IL-1ß maturation and release [11]. Although P2X4 closely correlates with P2X7, [12–16] the role of P2X4 in CS-driven pulmonary inflammation remains unclear.

In the present study, we demonstrate that *P2rx4* expression in the lungs of CS-exposed was increased. Furthermore, BALF cells and peripheral MNCs isolated from COPD patients, exhibit elevated *P2RX4* levels compared to healthy never smokers. Of note, whereas GOLD III and IV COPD patients show a remarkable increase in *P2RX4* levels, GOLD I and II COPD patients exhibit no significant *P2RX4* induction. Interestingly, active smoking tends to yield a higher *P2RX4* expression the BALF cells and blood MNCs compared to ex-smokers in the BALF cells and blood MNCs isolated from GOLD I and GOLD II patients. However, due to the pronounced differences in two known confounding factors, age and gender, between the groups as well as the small sample size, the results might be biased and we consider these human data rather exploratory. In order to draw a solid inference a larger number of volunteers with similar proportions of confounding factors is needed. Nevertheless, these data suggest that CS-exposure in mice as well as humans enhances *P2RX4* expression.

The inhibition as well as disruption of P2X4-signalling resulted in an alleviated CS-induced airway inflammation and proved functional relevance of the increased *P2rx4*-expression observed in CS-exposed mice. This is in line with previous studies reporting a suppressive effect of 5-BDBD treatment as well as *P2rx4*-deficiency in allergen-driven models of pulmonary inflammation [27, 28]. Besides a reduced airway inflammation, *P2rx4*-deficient mice showed less parenchymal destruction after four months of CS exposure accompanied by diminished neutrophil elastase as well as MMP9 BALF levels compared to wild type animals. Both proteases have been implicated in matrix remodelling and airway destruction in COPD [21]. These data suggest P2X4-signalling to contribute to CS-driven airway inflammation and airway remodelling.

In the airways, IL-1ß is considered a master regulator in immune defence against inhaled noxious including cigarette smoke [29]. BALF and sputum of COPD patients exhibit an increased IL-1ß production compared to never-smokers [30, 31]. Accordingly, mice overexpressing human IL-1ß show pulmonary inflammation and an increased secretion of CXCL1/KC, CXCL2/ MIP-2, MMP-9 and MMP-12 [9]. Interestingly, previous studies demonstrated that P2X4 regulates ATP-driven inflammasome activation and subsequent IL-1ß release in several organs [32]. Therefore, we suggested P2X4-signalling to contribute to CS-induced airway inflammation by promoting ATP-driven IL-1ß maturation and subsequent release. Support for this hypothesis comes from the reduced IL-1ß, CXCL1/KC and MMP-9 concentrations observed in the BALF of 5-BDBD treated as well as *P2rx*4-deficient mice in response to CS-exposure.

Among other cell types activated monocytes and macrophages are considered a major source of IL-1ß. In addition, myeloid cells play a crucial role in COPD pathogenesis and represent the predominant cell type in the BALF of smokers [22, 33]. Of note, monocytes and macrophages have been reported to exhibit a high P2RX4 expression according to www.proteinatlas.org/ and literature [23, 34]. We demonstrate that *P2RX4* expression, is considerably increased in COPD patients. In addition, non-polarized macrophages derived from the monocyte-like, human THP-1 cells pre-incubated with 5-BDBD released reduced amounts of not only IL-1ß, but other proinflammatory cytokines including IL-6, MCP-1 and TNFα in response to CSE stimulation.

It has been shown that P2X4 is preferentially localized in lysosomes of macrophages [35]. The intra-endolysosomal Ca^2+^ release triggered by activated P2X4 leads to calmodulin recruitment and the subsequent formation of P2X4-calmodulin complexes at endolysosomal membranes, which promote vesicle fusion [36]. Calcium/calmodulin binds to calcium-sensitive proteins like calmodulin dependent kinase II delta (CAMK2D), which have been implicated in modulating NF-κB signalling [37]. Active NF-κB, in turn, promotes the transcription of *proIL-1ß*, *proIL-18* and *Nlrp3*, all associated with inflammasome activation [38]. Therefore, we suggest that P2X4 promotes CS-induced airway inflammation by driving inflammasome activation, IL-1ß release and NF-κB activation in monocytic cells. However, P2X4-signalling in other cell types including hematopoietic and structural cells may also contribute to the noxious effects in the lung induced by CS. For instance, P2X4-signalling in airway epithelial cells as well as alveolar type II cells has been associated with an augmented mucin and surfactant secretion, and alveolar fluid transport [39, 40].

Besides P2X4, P2X7 has also been implicated in an ATP-dependent NF-κB activation [41–43]. Additionally, former studies suggest a physical interaction of P2X4 and P2X7 in myeloid cells [44]. Functionally, this interaction has been associated with ATP-induced IL-1ß release [15, 45]. Furthermore, both P2X4 and P2X7 have been implicated in NALP3 inflammasome activation [46–48]. Weinhold et al. propose compensatory mechanism of the expression of P2X4 and P2X7, thus knocking down the expression of one increases the expression of the other [16]. In contrast, several studies report that knocking out either P2X4 or P2X7 reduces the expression of the other one as well [14, 28]. While bone marrow derived macrophages (BMDMs) derived from wild type mice exhibit an increased *P2rx4* and *P2rx7* expression, *P2rx4*-deficient BMDMs fail to induce *P2rx7* in response to CSE stimulation (Additional file 1: Figs. S5, S6). Therefore, we suppose that P2X4-signalling modulates *P2RX7* expression in monocytic cells, which might augment suppressive effects associated with PX4-inhibition and *P2rx4-*deficiency.

## Conclusion

Taken together, our data demonstrate a clear role of P2X4 in cigarette smoke induced airway inflammation and cigarette smoke driven airway remodelling. The blocking of P2X4-signalling exerts inhibitory effects on immune cell lung recruitment and the subsequent release of proinflammatory cytokines and mediators including IL-1ß as well as proteases. This suggests that P2X4-signalling facilitates innate immunity in the lung and highlight the potential role of P2X4 in the course of immunopathologic responses induced by cigarette smoke. Therefore, the implications of these observations add another piece to the puzzle concerning the basic understanding of purinergic signalling in airway diseases.

## Supplementary Information

**Additional file 1.** Supplementary tables and figures.

## Data Availability

The datasets analyzed during the current study are available from the corresponding author on reasonable request.

## Abbreviations

5-BDBD: 5-(3-Bromophenyl)-1,3-dihydro-2H-benzofuro-[3,2-e]-1,4-diazepin-2-one
BALF: Bronchoalveolar lavage fluid
CS: Cigarette smoke
CSE: Cigarette smoke extract
ISA: Internal surface area
Lm: Mean linear intercept
P2rx4/P2RX4: Purinergic receptor P2X 4 (gene)
P2X4: Purinergic receptor P2X 4 (protein)
