# Supplementary material for: Blocking P2X purinoceptor 4 signalling alleviates cigarette smoke induced pulmonary inflammation
Source: Respir Res. 2022 Jun 8;23:148. doi: 10.1186/s12931-022-02072-z (PMC9175376; doi:10.1186/s12931-022-02072-z)
Supplement: Supplementary file 1 — Additional file 1. Supplementary tables and figures. [file 12931_2022_2072_MOESM1_ESM.pdf]

Blocking P2X Purinoceptor 4 Signalling Alleviates Cigarette Smoke-Induced Pulmonary Inflammation

Primer Sequences:

human *b2m*:	Forward:	5'-GATAGTTAAGTGGGATCGAGACATG-3'
	Reverse:	5'-AAAAGCAAGCAAGCAGAATTTGG-3'
	Probe:	5'-FAM-AATGCGGCATCTTCAAACCTCCATGAT-Tamra-3'
human *P2RX4*:	Forward:	5'-ATCCGCAGCCGCAAAGTG-3'
	Reverse:	5'-CACGGAGTCAGTTTCCTGGTAG-3'
	Probe:	5'-FAM-AATGCGGCATCTTCAAACCTCCATGAT-Tamra-3'
murine *b2m*:	Forward:	5'-GCAGAGTTAAGCATGCCAGTAT-3'
	Reverse:	5'-GGTTCAAATGAATCTTCAGAGCATC-3'
	Probe:	5'-FAM-TGATGCTTGATCACATGTCTCGATCCCAGTA-Tamra-3'
murine *P2rx4*:	Forward:	5'-AACACTTCTCAGCTTGGATTCCG-3'
	Reverse:	5'-TTCACGGTGACGATCATGTTGG-3'
	Probe:	5'-FAM-AGTTTTCCTCCTGAGCTGGGACCACATAGTC-Tamra-3'
murine *P2rx7*:	Forward:	5'-AAGTGGGTCTTGCACATGATC-3'
	Reverse:	5'-ATGCCTTTGACCTTGGTGTG-3'
	Probe:	5'-FAM-TCTTTTCCTACATTAGCTTTGCTTTGGTGAGC-Tamra-3'

Supplementary Table 1: Descriptive statistics of BALF donors

	GOLD I & II	GOLD III & IV	Total COPD patients	Non-smokers
No. of Patients	9	6	15	15
Age in years (mean ±SD)	62.2 ± 9.3	69.0 ± 5.3	64.9 ± 8.6	36.9 ± 11.3
Male in %	77.8	50.0	66.7	40.0
GOLD ratio	4 / 5	3 / 3	-	-

Supplementary Table 2: Descriptive statistics of MNCs donors

	GOLD I & II	GOLD III & IV	Total COPD patients	Non-smokers
No. of Patients	12	14	26	22
Age in years (mean ±SD)	58.8 ± 10.1	59.5 ± 6.6	59.2 ± 8.4	28.9 ± 4.3
Male in %	83.3	42.9	61.5	50.0
GOLD ratio	5 / 7	9 / 5	-	-

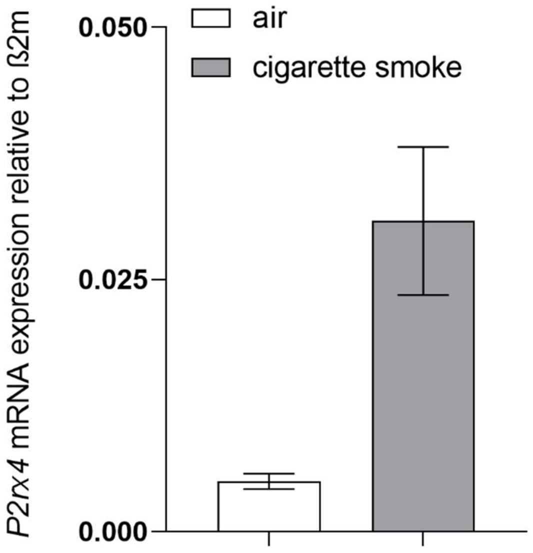

Supplementary Figure 1

Relative *P2rx4* expression in pooled BALF cells from 6 wild type mice per group. The *P2rx4* mRNA expression is quantified relative to ß2 microglobulin mRNA (*ß2m*) levels and presented as mean ±SD of the triplicate assay.

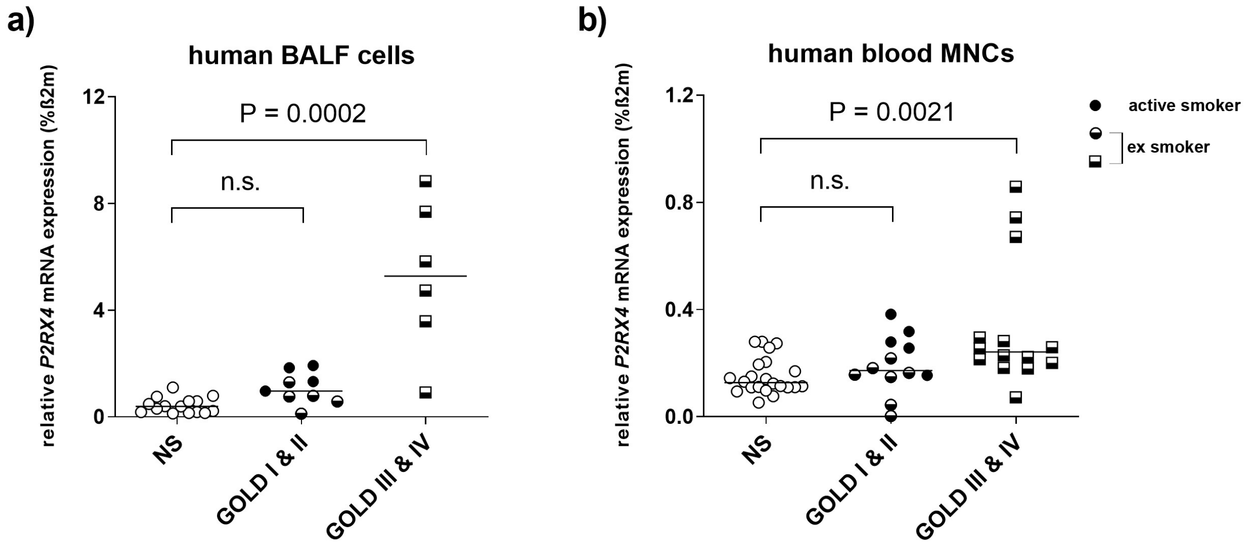

Supplementary Figure 2

Relative *P2RX4* expression in a) BALF cells and b) peripheral blood MNCs of COPD patients stratified for GOLD classification into mild-moderate (GOLD I & II) and severe (GOLD III & IV) and compared to healthy never-smoking individuals (NS). P2X4 mRNA expression is quantified relative to ß2 microglobulin mRNA (*ß2m*) levels. Median expression levels are presented as horizontal lines. Each symbol represents a subject (BALF: n=6-15, monocytes: n=12-22). Shapes completely black represent current smokers and half-filled shapes represent the ex-smokers among the COPD patients. Data was analyzed using Kruskal-Wallis test with Dunn's post-hoc test to correct for multiple comparison. P is depicted in the corresponding graph and was considered statistical significant when ≤0.5. n.s. = non-significant

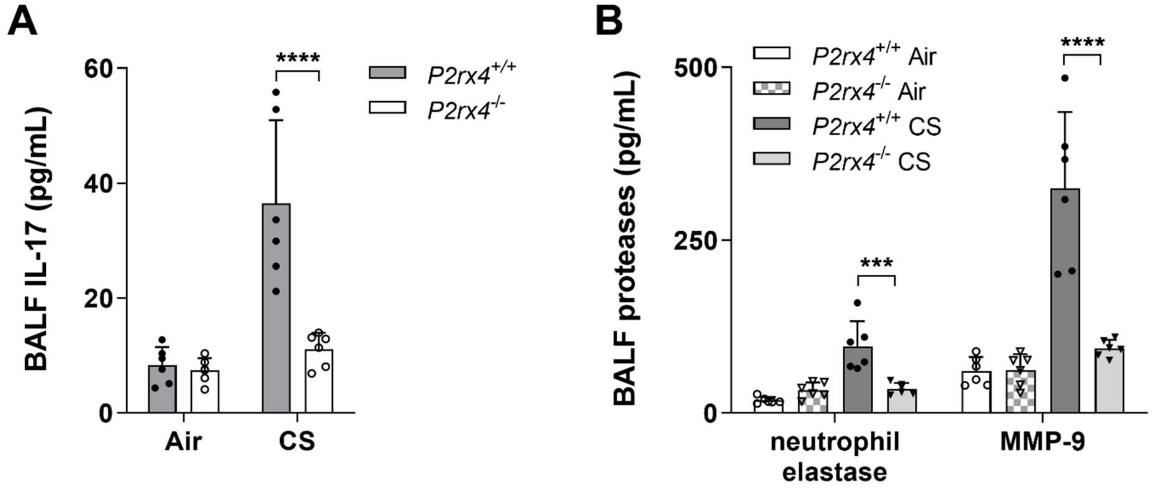

Supplementary Figure 3

Concentration of A) IL-17, B) neutrophil elastase and MMP-9 in the BALF of *P2rx4*-deficient mice (*P2rx4*$^{-/-}$) after cigarette smoke (CS) exposure for three consecutive days compared to wild type animals (*P2rx4*$^{+/+}$). Each animal is represented by an individual symbol (n=6). Data is presented as mean +SD and was analysed applying two-way ANOVA corrected for multiple comparison using Tukey test. *** P<0.001, **** P<0.0001

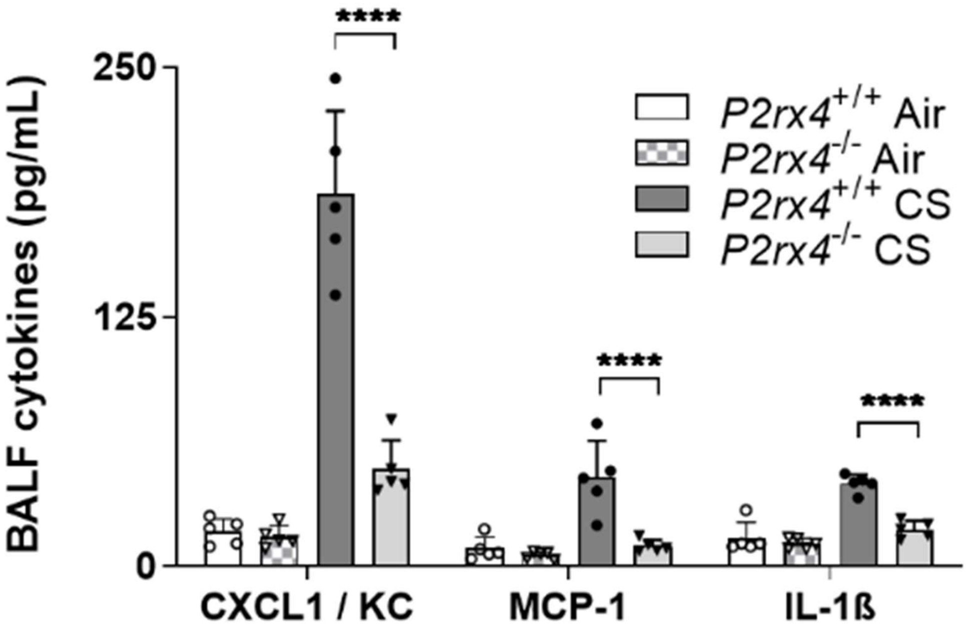

Supplementary Figure 4

Concentration of proinflammatory cytokines including CXCL1 / KC, IL-1ß and IL-6 in the BALF of *P2rx4*-deficient mice (*P2rx4*$^{-/-}$) after exposure with the smoke of 5 cigarettes per day, five days a week for four months compared to wild type animals (*P2rx4*$^{+/+}$). Each animal is represented by an individual symbol (n=5). Data is presented as mean +SD and was analysed applying two-way ANOVA corrected for multiple comparison using Tukey test. **** P<0.0001

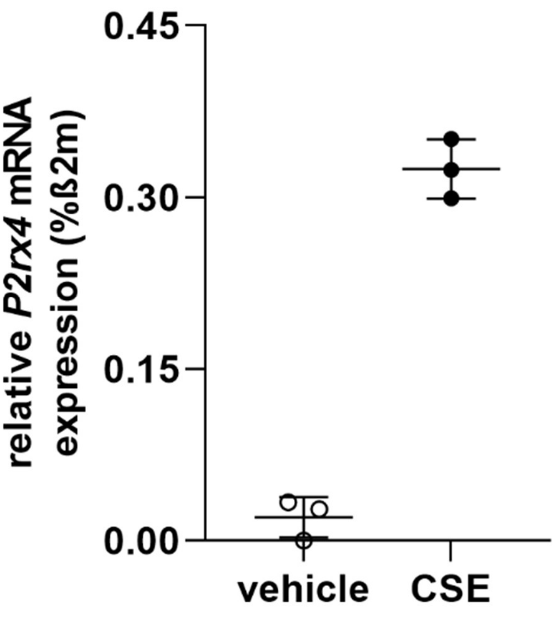

Supplementary Figure 5

P2rx4 expression quantified relative to ß2 microglobulin mRNA (ß2m) levels of bone marrow derived macrophages isolated from either wild type mice after vehicle or 5 % cigarette smoke extract stimulation. Mean ± SD expression levels of the triplicate assay are presented as horizontal lines and the range is indicated by the error bars.

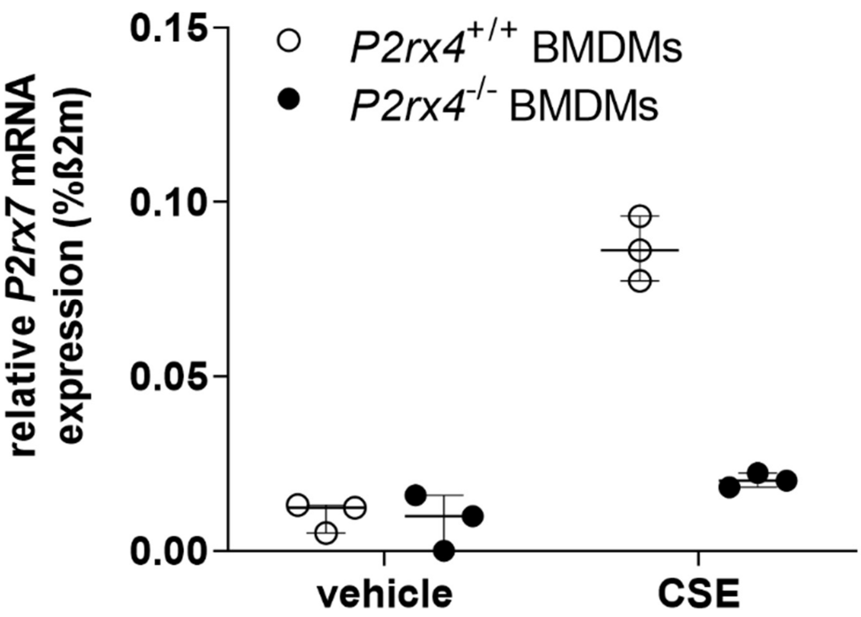

Supplementary Figure 6

P2rx7 expression quantified relative to ß2 microglobulin mRNA (ß2m) levels of bone marrow derived macrophages isolated from either *P2rx4*-deficient or wild type mice after vehicle or 5 % cigarette smoke extract stimulation. Mean ± SD expression levels of the triplicate assay are presented as horizontal lines and the range is indicated by the error bars.
